# Neural Pathways of Stress Integration: Relevance to Alcohol Abuse

**DOI:** 10.35946/arcr.v34.4.08

**Published:** 2012

**Authors:** James P. Herman

**Affiliations:** **James P. Herman, Ph.D.,***is a professor in the Departments of Psychiatry and Behavioral Neuroscience, University of Cincinnati, Cincinnati, Ohio.*

**Keywords:** Addiction, alcohol and other drug–seeking behavior, alcohol use and abuse, stress, stressor, chronic stress reaction, stress integration, physiological response to stress, psychogenic stress responses, brain, neural pathways, limbic-paraventricular pathway, limbic stress control network, hypothalamic–pituitary–adrenal axis, literature review

## Abstract

Stress is a critical component in the development, maintenance, and reinstatement of addictive behaviors, including alcohol use. This article reviews the current state of the literature on the brain’s stress response, focusing on the hypothalamic–pituitary–adrenal (HPA) axis. Stress responses can occur as a reaction to physiological (or systemic) challenge or threat; signals from multiple parts of the brain send input to the paraventricular nucleus (PVN) within the hypothalamus. However, responses also occur to stressors that predict potential threats (psychogenic stressors). Psychogenic responses are mediated by a series of nerve cell connections in the limbic–PVN pathway, with amygdalar and infralimbic cortex circuits signaling excitation and prelimbic cortex and hippocampal neurons signaling stress inhibition. Limbic–PVN connections are relayed by predominantly GABAergic neurons in regions such as the bed nucleus of the stria terminalis and preoptic area. Chronic stress affects the structure and function of limbic stress circuitry and results in enhanced PVN excitability, although the exact mechanism is unknown. Of importance, acute and chronic alcohol exposure are known to affect both systemic and psychogenic stress pathways and may be linked to stress dysregulation by precipitating chronic stress–like changes in amygdalar and prefrontal components of the limbic stress control network.

Adaptation in the face of physical or psychological adversity is required for the survival, health, and well-being of all organisms. Adverse events, often denoted as “stressors,” initiate a diverse physiological response from multiple sources, including activation of the hypothalamic–pituitary–adrenal (HPA) axis.^1^ The HPA axis is responsible for the glucocorticoid component of the stress response (i.e., steroid hormone response; cortisol in humans, corticosterone in mice and rats). Glucocorticoid secretion is thought to contribute to stress adaptation by causing long-term changes in gene expression via cognate adrenocorticosteroid receptors (i.e., mineralocorticoid receptor [MR] and glucocorticoid receptor [GR]). The adrenocorticosteroid receptors function as ligand-gated transcription factors (De Kloet et al. 1998) but can also modulate transcription by interfering with other transcriptional regulators, such as nuclear factor-κB (NF-κB) and activator protein-1 (AP-1) (Webster and Cidlowski 1999). Glucocorticoids also can have rapid effects on brain chemistry and behavior via nongenomic membrane signaling mechanisms (De Kloet et al. 2008). Glucocorticoids are thought to contribute to termination of the initial stress response (Keller-Wood and Dallman 1984) and to participate in long-term restoration of homeostasis triggered by the initial response (Munck et al. 1984).

Glucocorticoid stress responses can be initiated by physiological perturbations (representing reflexive responses) or by brain processes linking environmental cues with probable negative outcomes. The latter so-called “psychogenic” response is anticipatory in nature and involves brain pathways responsible for innate defense programs or memory of aversive events (Herman et al. 2003). Thus, the psychogenic response is related to prior experience, and it is designed to energetically prepare the organism to either avoid an adverse outcome or engage in behaviors that can maximize the potential for survival.

Considerable evidence indicates that stress systems play a major role in addictive processes, including alcohol dependence. For example, exposure to stress can precipitate relapse or increase alcohol use (Sinha 2007). Actions of stress/glucocorticoids on alcohol intake can be linked to modulation of reward/stress circuitry, including, for example, enhancement of dopamine release in the nucleus accumbens (Sutoo and Akiyama 2002; Yavich and Tiihonen 2000) and activation of central corticotropin-releasing factor (CRF) pathways (Heilig and Koob 2007). Notably, the link between alcohol intake and stress is complicated by the fact that exposure to alcohol, like many drugs of abuse, causes the release of glucocorticoids upon exposure and thus can be classified as an acute “stressor” of sorts (see Allen et al. 2011).

This article reviews the organization of neurocircuits that regulate stress responses, focusing on the HPA axis, which is of particular relevance to addictive processes (see Marinelli and Piazza 2002). It also discusses areas of intersection between stress and reward pathways, as these are likely important in mediating the deleterious effects of stress on substance abuse and addiction.

## Circuitry Mediating the Reflexive Stress Response

**Figure 1:**
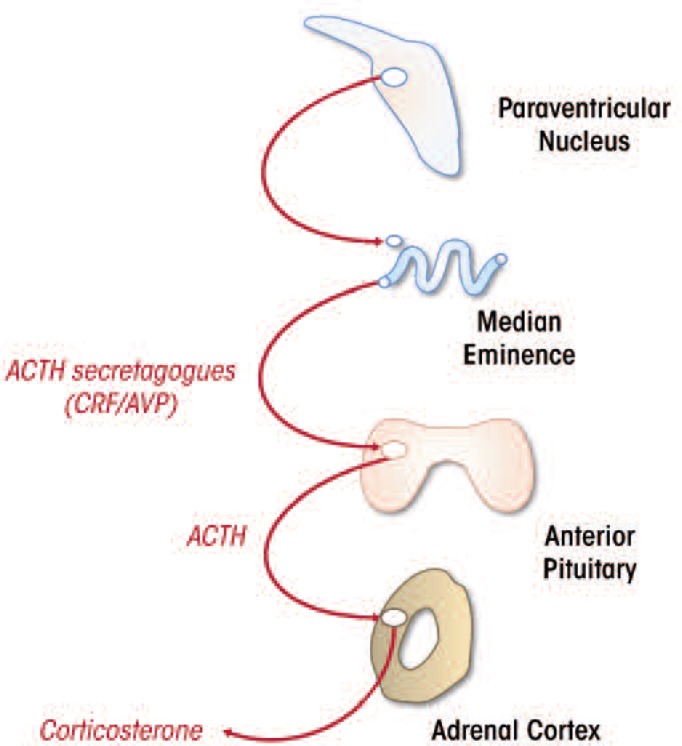
Schematic of the hypothalmic–pituitary–adrenal (HPA) axis of the rat. HPA responses are initiated by neurosecretory neurons of medial parvocellular paraventricular nucleus (mpPVN), which secretes adrenocorticotropin (ACTH) secretagogues such as corticotropin-releasing factor (CRF) and arginine vasopressin (AVP) in the hypophysial portal circulation at the level of the median eminence. These secretagogues promote release of ACTH into the systemic circulation, whereby it promotes synthesis and release of glucocorticoids at the adrenal cortex.

The HPA axis is controlled by neurons within the paraventricular nucleus (PVN) in the hypothalamus (see figure 1). These neurons secrete CRF and the hormone vasopressin into the portal circulation, which then triggers the release of adrenocorticotropin hormone (ACTH) from the anterior pituitary gland. ACTH travels via the systemic circulation to reach the adrenal cortex, wherein glucocorticoids are synthesized and released (see Herman et al. 2003).

Reflexive stress responses occur during emergencies (e.g., infection, starvation, dehydration, or shock), when the brain must respond to a substantial challenge to homeostasis by mobilizing the HPA axis. Sensory information is communicated to the PVN by first- or second-order neurons, generating a direct activation of CRF release (see Herman et al. 2003). For example, low blood pressure associated with blood loss is relayed via sensory nerves to brainstem neurons in the A2 catecholaminergic cell group (Palkovits and Zaborszky 1977), which then project directly to the PVN (Cunningham and Sawchenko 1988) and rapidly elicit noradrenergic activation of CRF neurons (Plotsky et al. 1989).

In addition to neural pathways, information on changes in physiological state also may be relayed via circulating factors that bind to areas outside the blood–brain barrier. For example, peripheral increases in the hormone angiotensin II (signaling dehydration) are sensed by receptors in the subfornical organ (which is located outside the blood–brain barrier and regulates fluid balance), which sends direct angiotensin II projections to the PVN CRF neurons, facilitating HPA activation (Plotsky et al. 1988). Some peripheral stimuli, such as inflammation, produce factors that can signal by multiple mechanisms; for example, the proinflammatory cytokine interleukin 1-b seems to activate the HPA axis via sensory nerve fibers in the vagus nerve; the area postrema, which is outside the blood–brain barrier; and perivascular cells in the region of the A2 cell group (Ericsson et al. 1997; Lee et al. 1998; Wieczorek and Dunn 2006).

Drugs of abuse also may produce an initial corticosterone response via brainstem PVN-projecting pathways. For example, initial exposure to alcohol causes ACTH and corticosterone release, consistent with alcohol acting as an unconditioned stimulus (Allen et al. 2011). Acute HPA axis activation by alcohol is mediated by brainstem noradrenergic systems (Allen et al. 2011). However, chronic exposure to alcohol significantly blunts HPA axis activation to acute alcohol exposure (Rivier 1995), suggesting that, to some degree, direct HPA excitatory effects of alcohol use habituate over time.

## Circuitry Subserving Anticipatory Stress Responses: The Limbic Stress-Control Network

Because true physiologic “emergencies” are relatively rare, the vast majority of stress responses are anticipatory in nature, involving interpretation of the threat potential of environmental stimuli with respect to previous experience or innate programs. Anticipatory stress responses are largely controlled by limbic forebrain structures, such as the hippocampus, medial prefrontal cortex (mPFC), and amygdala (see Ulrich-Lai and Herman 2009). These structures all receive processed sensory information and are involved in regulation of emotion, reward, and mood.

Brain lesion and stimulation studies indicate that the hippocampus inhibits the HPA axis. Electrical stimulation of the hippocampus decreases glucocorticoid release in rats and humans. Damage to the hippocampus, or the nerves carrying impulses away from it (i.e., lateral fornix), cause exaggerated responses to psychogenic stressors (e.g., restraint) and manifest as a prolonged return to baseline glucocorticoid levels (for primary references, see Herman et al. 2003; Jacobson and Sapolsky 1991). Some data suggest that the hippocampus also inhibits basal HPA axis activity, but this is not universally observed (Herman et al. 2003; Jacobson and Sapolsky 1991). The effects of hippocampal damage on psychogenic HPA axis stress responses can be localized to the ventral subiculum (vSUB), the main subcortical output of the ventral hippocampus (Herman et al. 2003). Discrete lesions of the vSUB in rats enhance PVN CRF peptide and mRNA expression and increase corticosterone release and PVN activation (as determined by induction of FOS mRNA expression) in response to restraint (Herman et al. 1998).

The effect of the vSUB on stress regulation is stressor specific. Lesions of the vSUB prolong HPA axis responses to novelty but do not affect reflexive responses (e.g., to ether inhalation) (Herman et al. 1998). Some evidence suggests that glucocorticoids play a role in hippocampal inhibition of anticipatory responses, as lesions can block feedback inhibition of the HPA axis by the synthetic steroid dexamethasone (Magarinos et al. 1987). In addition, mice with forebrain GR deletions, including the hippocampus, have exaggerated responses to restraint and novelty (but not hypoxia) and impaired dexamethasone suppression of corticosterone release (Boyle et al. 2005; Furay et al. 2008). Together, the data indicate that the hippocampus is specifically engaged in regulation of responses to psychogenic stressors, in keeping with its role in cognitive processing and emotion.

Unlike the hippocampus, the amygdala is associated with excitation of the HPA axis. Amygdalar stimulation promotes glucocorticoid release, whereas large lesions of the amygdaloid complex reduce HPA axis activity (see Herman et al. 2003). However, there is a marked subregional specialization of stress-integrative functions within the amygdala. The central nucleus of the amygdala (CeA) is highly responsive to homeostatic stressors, such as inflammation and blood loss (Dayas et al. 2001; Sawchenko et al. 2000). Lesions of the CeA attenuate HPA axis responses to these types of stimuli but not to restraint (Dayas et al. 1999; Prewitt and Herman 1997; Xu et al. 1999). In contrast, the medial nucleus of the amygdala (MeA) shows preferential FOS responses to stimuli, such as restraint (Dayas et al. 2001; Sawchenko et al. 2000). Lesions of the MeA reduce HPA axis responses to restraint and light and sound stimuli but not to systemic injection of the protein interleukin 1-b or ether inhalation (Dayas et al. 1999; Feldman et al. 1994). Thus, it seems that reflexive and anticipatory responses may be regulated in part by discrete amygdaloid circuitry.

The mPFC seems to have a complex role in stress regulation. All divisions of the rodent PFC are robustly activated by acute stress. However, the physiological consequences of stress activation seem to vary by region. The prelimbic division of the mPFC (PL) is important in stress inhibition because numerous studies have shown that damage to this region prolongs HPA axis responses to acute psychogenic (but not homeostatic) stressors (Diorio et al. 1993; Figueiredo et al. 2003; Radley et al. 2006), whereas stimulation inhibits stress responses (Jones et al. 2011). The mPFC seems to be a site for glucocorticoid feedback of HPA responses because local glucocorticoid implants inhibit anticipatory (but not reflexive) responses to stressors (Akana et al. 2001; Diorio et al. 1993). In contrast, lesions directed at the more ventral infralimbic PFC (IL) have a markedly different physiological effect. Damage to the IL decreases autonomic responses to psychogenic stressors (Tavares et al. 2009) and also attenuates PVN FOS activation in response to restraint (Radley et al. 2006). Thus, the PL and IL seem to have opposing effects on stress integration.

## Running the Relay: Limbic–PVN Networks

**Figure 2:**
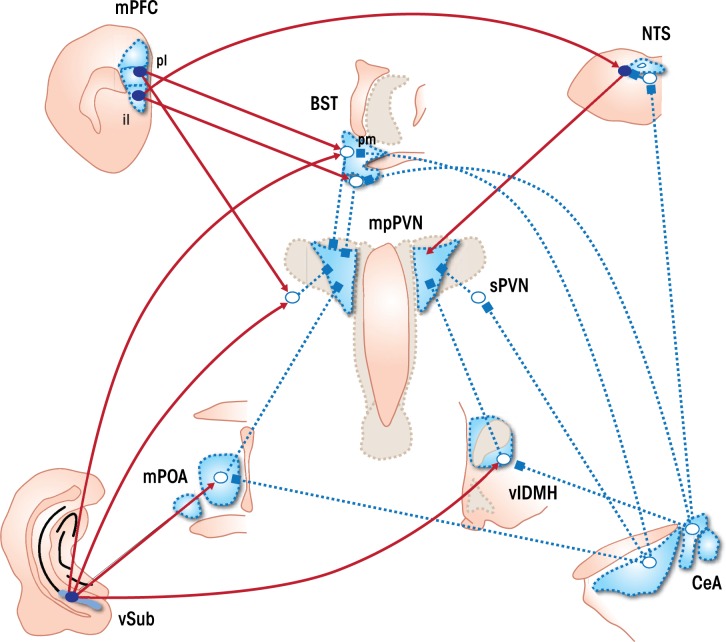
Schematic of limbic stress-integrative pathways from the prefrontal cortex, amygdala and hippocampus. The medial prefrontal cortex (mPFC) subsumes neurons of the prelimbic (pl) and infralimbic cortices (il), which appear to have different actions on the hypothalmic–pituitary–adrenal (HPA) axis stress response. The pl sends excitatory projections (designated as dark circles, filled line with arrows) to regions such as the peri-PVN (peri-paraventricular nucleus) zone and bed nucleus of the stria terminalis (BNST), both of which send direct GABAergic projections to the medial parvocellular PVN (delineated as open circles, dotted lines ending in squares). This two-neuron chain is likely to be inhibitory in nature. In contrast, the infralimbic cortex projects to regions such as the nucleus of the solitary tract (NTS) and the anterior BNST, which sends excitatory projections to the PVN, implying a means of PVN excitation from this cortical region. The ventral subiculum (vSUB) sends excitatory projections to numerous subcortical regions, including the posterior BNST, peri-PVN region (including the subparaventricular zone [sPVN], medial preoptic area [POA] and ventrolateral region of the dorsomedial hypothalamic nucleus [vlDMH]), all of which send GABAergic projections to the PVN and are likely to communicate transsynaptic inhibition. The medial amygdaloid nucleus (MeA) sends inhibitory projections to GABAergic PVN-projecting populations, such as the BNST, POA and sPVN, eliciting a transsynaptic disinhibition. A similar arrangement likely exists for the central amygdaloid nucleus (CeA), which sends GABAergic outflow to the ventrolateral BST and to a lesser extent, the vlDMH. The CeA also projects to GABAergic neurons in the NTS, which may disinhibit ascending projections to the PVN.

Stimulation of the PVN by the hippocampus, prefrontal cortex, and amygdala is quite limited. Therefore, regulation of HPA axis output by these structures requires intermediary synapses (see figure 2). Studies that trace projections from one part of the brain to another (i.e., tract-tracing studies) reveal the potential for bisynaptic limbic–PVN connections traversing a number of subcortical regions, including the bed nucleus of the stria terminalis (BNST), dorsomedial hypothalamus, medial preoptic area, and peri-PVN region (including the subparaventricular nucleus) (Cullinan et al. 1993; Prewitt and Herman 1998; Vertes 2004). Dual-tracing studies indicate that nerves carrying impulses away from the vSUB, MeA, and CeA (i.e., efferent nerves) directly contact PVN-projecting neurons in these regions, consistent with functional interconnections (Cullinan et al. 1993; Prewitt and Herman 1998).

The differential effects of PL and IL on stress effector systems may reflect their marked divergence in subcortical targets. The PL has substantial projections to reward-relevant pathways, including the nucleus accumbens and basolateral amygdala, as well as the posterior BNST, which is linked to HPA axis inhibition. In contrast, the IL has rich interconnections with regions involved in autonomic regulation, including the CeA, nucleus of the solitary tract (NTS), anteroventral BNST, and dorsomedial hypothalamus (Vertes 2004). Thus, it is probable that the net effect of PFC stress activation requires subcortical integration of PL and IL outflow.

Of note, mPFC, hippocampal, and amygdalar efferents tend to be concentrated in regions sending γ-aminobutyric acid (GABA)-carrying projections to the PVN (see figure 2). Indeed, the vast number of sub-innervated PVN-projecting neurons are GABAergic in phenotype. Projection neurons of the vSUB (as well as the mPFC) are glutamatergic in nature, thus suggesting that these cells engage in transsynaptic inhibition of the PVN following activation by stress. In contrast, the projection neurons of the MeA and CeA are predominantly GABAergic, suggesting that amygdalar excitation of the PVN is mediated by disinhibition, involving sequential GABA synapses (Herman et al. 2003).

The BNST is of particular interest, in that it receives inputs from all of the major limbic stress-integrative structures (CeA, MeA, vSUB, IL, and PL) (Cullinan et al. 1993; Dong et al. 2001; Vertes 2004). Of note, different BNST subregions seem to be responsible for inhibition versus excitation of HPA axis stress responses. For example, lesions of the posterior medial region of the BNST increase the magnitude of ACTH and corticosterone release and PVN FOS activation (Choi et al. 2007), implying a role in central integration of stress inhibition. Lesions of the anteroventral component of the BNST also enhance stress responses (Radley et al. 2009). In contrast, larger lesions of the anterior BNST reduce HPA axis stress responses (Choi et al. 2007), consistent with a role for this region in stress excitation. Thus, the role of the BNST in stress inhibition versus activation is compartmentalized and may be associated with differences in limbic targeting of individual subregions of the BNST. For example, the posterior medial BNST receives heavy innervation from the vSUB and MeA, whereas the anteroventral region receives input from the CeA and most of the IL efferents (Canteras and Swanson 1992; Cullinan et al. 1993; Dong et al. 2001; Vertes 2004).

The medial preoptic area and peri-PVN regions are heavily populated with GABAergic neurons and seem to primarily modulate stress inhibition (Herman et al. 2003). Neurons in these regions are believed to provide tonic inhibition to the PVN, which can be adjusted in accordance with glutamate inputs from the vSUB (enhanced inhibition) or GABAergic inputs primarily from the MeA (disinhibition). Lesions of the medial preoptic nucleus increase HPA axis stress responses and block HPA axis responses elicited by medial amygdalar stimulation, suggesting a primary role in stress inhibition (for primary references, see Herman et al. 2003). Local inhibition of glutamate signaling in the peri-PVN region also enhances HPA axis stress responses (Ziegler and Herman 2000), suggesting that limbic axons terminating in this region may modulate PVN activation.

It is more difficult to pinpoint the role of other hypothalamic regions linking limbic efferents to the PVN, such as the dorsomedial nucleus (Herman et al. 2003). For example, conflicting results are observed following lesion, activation, or inactivation of this dorsomedial hypothalamus, possibly because of heavy mixing of glutamate and GABA neuronal populations (Herman et al. 2003).

Additional potential relays remain to be fully explored. For example, the raphe nuclei and NTS innervate the PVN, are targeted by limbic structures (such as the PL) (see Vertes 2004) and are involved in stress excitation by serotonin and norepinephrine (Herman et al. 2003), respectively. However, as yet, there are no anatomical studies describing bisynaptic limbic–PVN relays through these regions.

## Circuitry Subserving Chronic Stress Responses

Prolonged or extended exposure to stress causes long-term upregulation of the HPA axis, characterized by reduced thymus weight (attributed to cumulative elevations in GCs); increased adrenal size (attributed to increased ACTH release); increased adrenal sensitivity to ACTH; facilitated HPA axis responses to novel stressors; and in some (but not all) paradigms/conditions, elevated basal GC secretion (see Herman et al. 1995; Ulrich-Lai et al. 2006). Changes in peripheral hormone release are accompanied by increased PVN CRF and vasopressin mRNA (Herman et al. 1995), suggesting that HPA upregulation is centrally mediated. In addition, chronic stress increases glutamatergic and noradrenergic terminal abutting PVN CRF neuronal somata and dendrites, consistent with enhanced excitatory synaptic drive (Flak et al. 2009).

Central mechanisms of chronic HPA axis activation have yet to be determined. The role of the limbic forebrain in stress control suggests that differential involvement of the PFC, hippocampus, and amygdala may be responsible for prolonged drive. Of note, all regions show significant chronic stress–induced neuroplastic changes: Dendritic retraction is evident in hippocampal and mPFC pyramidal neurons, whereas dendritic extension is observed in the amygdala (for primary references, see Ulrich-Lai and Herman 2009). These studies are consistent with redistribution of limbic input to HPA excitatory circuits, favoring excitation over inhibition.

Enhanced amygdalar drive is proposed to play a major role in chronic stress pathology. For example, chronic stress activates the CeA CRF system, which has been proposed as a chronic stress–recruited pathway (Dallman et al. 2003). However, the CeA does not seem to be required for the development or maintenance of chronic stress symptoms (Solomon et al. 2010). In addition, lesions of the MeA also fail to prevent chronic stress drive of the HPA axis (Solomon et al. 2010). Thus, the overall link between amygdalar hyperactivity and chronic stress–induced HPA axis dysfunction has yet to be firmly established.

The paraventricular nucleus of the hypothalamus (PVT) seems to comprise a component of the chronic-stress pathway. Lesions of the PVT block chronic stress sensitization of HPA axis responses to novel stressors (Bhatnagar and Dallman 1998), suggesting a primary role in the facilitation process. In addition, PVT lesions disrupt the process of HPA axis habituation to repeated stressors (Bhatnagar et al. 2002). Taken together, the data suggest the PVT plays a major role in gating HPA axis drive in the context of prolonged stress exposure. Of note, the PVT and limbic forebrain sites that control acute stress responses are interconnected (see Vertes and Hoover 2008), allowing for possible coordination of corticolimbic stress outputs in this region. The PVT also is positioned to process information regarding ongoing physiological status, receiving inputs from orexinergic neurons (which regulate the release of acetylcholine, serotonin, and noradrenaline) of the dorsolateral hypothalamus (which plays an integral role in control of arousal processes) and ascending brainstem systems involved in autonomic control.

The BNST also is positioned to integrate information on chronic stress. Lesions of the anteroventral BNST attenuate responses to acute stress, but potentiate facilitation of the HPA axis by chronic stress (Choi et al. 2008). These data suggest that this region has chronicity-dependent roles in HPA axis control, with presumably different neural populations recruited in an attempt to respond to prolonged stress exposure. Given intimate interconnectivity between the anterior BNST and mPFC, hippocampus, and amygdala, it is possible that BNST neurons may be “reprogrammed” by chronic stress–induced changes in limbic activity or innervation patterns.

## Stress Circuitry and Alcohol

Readers familiar with the alcohol literature will no doubt find considerable overlap between the stress circuitry described above and brain circuitry linked to alcohol intake. For example, considerable data support a role for the CeA, BNST, and noradrenergic systems in the maintenance of alcohol dependence (see Koob 2009), suggesting that the process of addiction is linked to activation of stress (and HPA axis) excitatory pathways. Indeed, enhanced CeA/BNST CRF expression resembles what would be expected after chronic stress, leading to the hypothesis that negative addictive states (e.g., avoidance of withdrawal) are linked to alcohol-induced recruitment of chronic stress circuits (Koob 2009). Conversely, activation of reward pathways is known to significantly buffer stress reactivity via the amygdaloid complex, suggesting a mechanism whereby the rewarding effects of alcohol may reduce perceived stress (Ulrich-Lai et al. 2010).

Alcohol also has profound effects on medial prefrontal cortical neural activity, and chronic use is associated with prefrontal hypofunction (poor impulse control) in humans (see Abernathy et al. 2010). The mPFC projects to both the CeA and BNST and, at least in the case of the prelimbic region, plays a prominent role in HPA inhibition. In combination with the gain of function seen in amygdalar–BNST circuits, these observations suggest that chronic alcohol use causes marked changes across the limbic stress control network, biasing the organism for stress hyperreactivity.

Overall, adequate control of the HPA axis is a requirement for both short- and long-term survival. Given that key control nodes of HPA axis activity are targeted by alcohol, and that alcohol itself constitutes a threat, it is not surprising that corticosteroids, the “business end” of the axis, have profound interactions with both behavioral and physiological regulation of intake. The overlap between HPA regulatory and addiction circuits identifies key points that may be targets for both the long-term detrimental effects of alcohol abuse as well as dependence itself. The importance of circuit overlap is further underscored by the powerful reciprocal relationship between life stress and drinking, which complicates efforts to establish and maintain abstinence.

## Footnotes

1 For the definition of this and other technical terms, see the Glossary, pp. 522–524.
